# Nontuberculous Mycobacterial Infection in Patients with Neurosurgical Hardware: Two Cases and A Review of the Literature

**DOI:** 10.7759/cureus.7398

**Published:** 2020-03-24

**Authors:** Varun Padmanaban, Rezhan Hussein, Elias Rizk

**Affiliations:** 1 Neurological Surgery, Penn State Milton S. Hershey Medical Center, Hershey, USA; 2 Medicine/Infectious Disease, Penn State Milton S. Hershey Medical Center, Hershey, USA

**Keywords:** nontuberculous mycobacteria, cns infection, ventriculoperitoneal shunt, case report, review

## Abstract

Central nervous system infections with nontuberculous mycobacteria (NTM) are rare but have been increasing in frequency. A small fraction of these infections are related to surgical hardware, with approximately 20 cases reported. Patients typically present with an indolent course but can rapidly deteriorate. We report two novel cases of NTM infection in ventriculoperitoneal shunts, and review the literature on treatment options, challenges and outcomes in these patients. Clinicians should consider NTM when dealing with unusual hardware infections as it is an emerging infectious disease with high potential for morbidity and mortality.

## Introduction

Nontuberculous mycobacteria (NTM) defined as mycobacterial species other than Mycobacterium tuberculosis complex or Mycobacterium leprae. These organisms are ubiquitous and are found in water, dust and soil. Reports of infection with the organisms have been increasing worldwide, involving the lungs, skin, soft tissue and central nervous system [1]. Central nervous system (CNS) infections with NTM are rare but have high mortality.

Cerebral spinal fluid (CSF) infection from neurosurgical hardware is relatively common with rates ranging from 5% to 15% [2]. Most infections develop from colonization of skin flora [2]. We report two cases of NTM infection in neurosurgical patients related to ventriculoperitoneal (VP) shunt hardware, and review the literature, common presentations and current treatment strategies for CNS NTM infections related to neurosurgical hardware.

## Case presentation

Patient no. 1

A 24-year-old man with a history of posterior fossa tumor requiring VP shunting presented with worsening headaches, confusion and weight loss over several months. He had a history of vague pain along the shunt site, chest and abdomen as well as swallowing difficulties over several weeks. The patient had a history of multiple shunt revisions, the most recent of which approximately 10 months prior to our evaluation when he had undergone a proximal shunt revision for an obstructed ventricular catheter.

Patient Course

**Figure 1:**
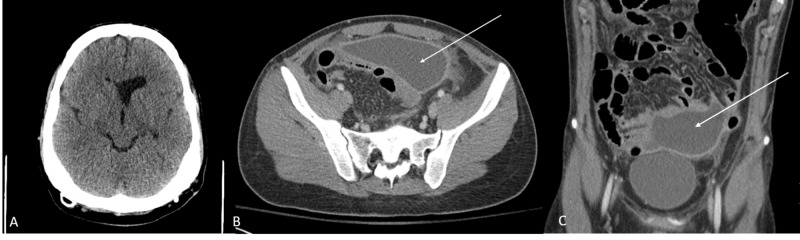
CT without contrast of head and abdomen CT head without contrast (A) showing stable ventricular size on admission. CT abdomen without contrast showing abdominal pseudocyst (arrow) on axial (B) and coronal (C) sequences.

A CT of the head and abdomen revealed ventricles stable in size and a pseudocyst around his distal catheter (Figure 1).

**Figure 2:**
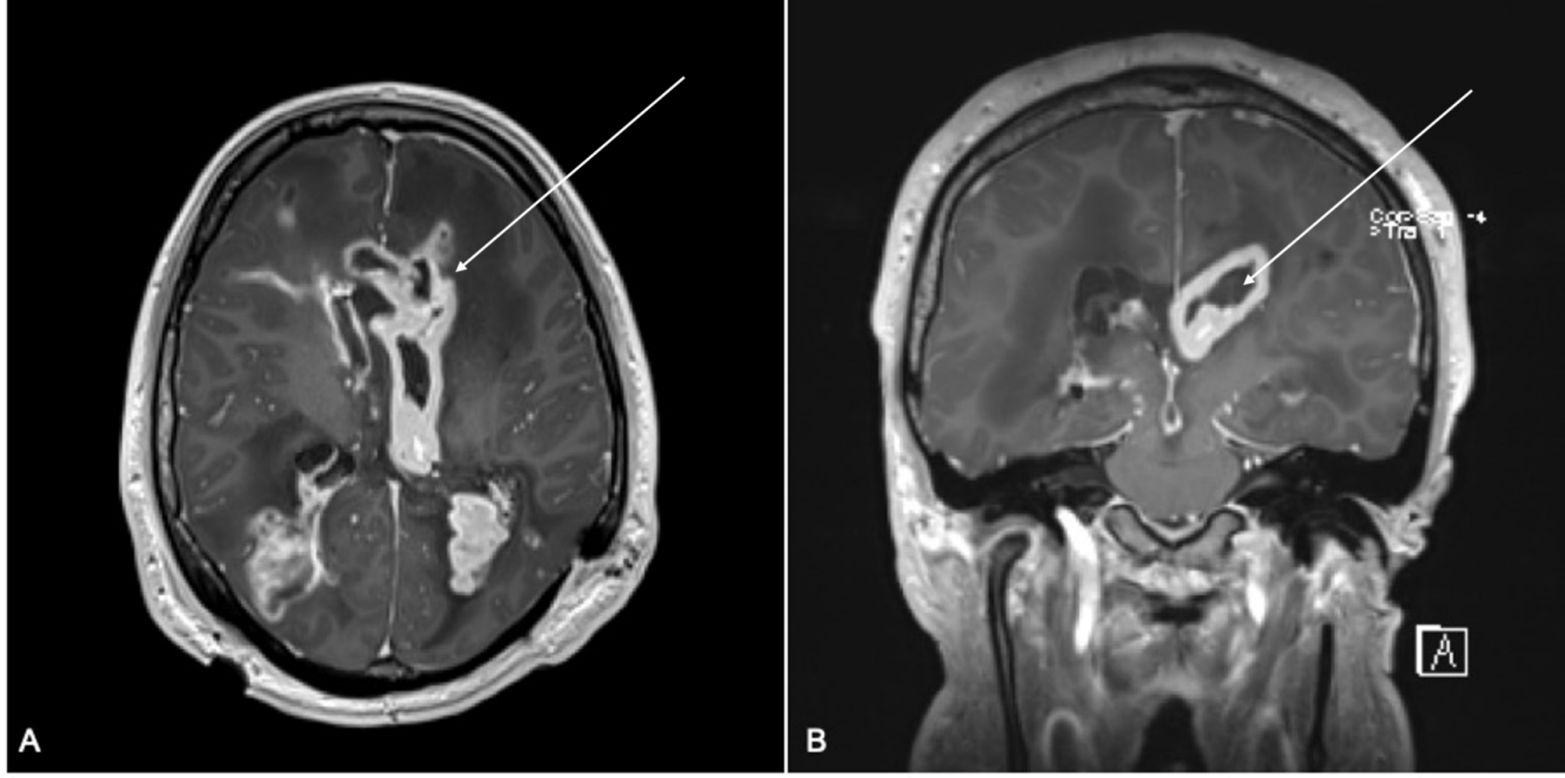
MRI T1 with contrast MRI T1 with contrast showing severe ventriculitis (arrow) and adjacent cerebral inflammatory changes with edema in axial (A) and coronal (B) sequences.

He was immediately taken to the operating room for complete shunt externalization and placement of an external ventricular drain (EVD). He was started on broad-spectrum antibiotics (vancomycin, cefepime and metronidazole) and underwent percutaneous drainage of the pseudocyst in his abdomen. Cultures from both the percutaneous drainage and CSF were positive for acid-fast bacilli (AFB) and presumed NTM. His antibiotics were switched to azithromycin, amikacin and cefoxitin by postadmission day 4. He continued to decompensate requiring multiple EVD replacements due to loculated ventriculitis. AFB was identified as Mycobacterium abscesses. Intrathecal (IT) amikacin was started on postadmission day 9. This was delayed due to the inability to clamp the EVD and safely administer IT amikacin. During the course of treatment, he had significant QTc interval prolongation which necessitated holding IV azithromycin and starting linezolid with readministration of azithromycin via nasogastric tube to minimize the risk of QTc prolongation by avoiding high peaks. He remained critically ill on a ventilator and required 12 EVD replacements in attempts to clear his loculated hydrocephalus. A brain MRI with contrast showed severe ventriculitis and cerebral inflammation (Figure 2). 

On postadmission day 30, he was transitioned to comfort care. He passed away on postadmission day 36.

Patient no. 2

A 71-year-old woman with normal pressure hydrocephalus status post placement of VP shunt approximately two years prior presented with recurrent and prolonged abdominal discomfort. She had a history of multiple abdominal surgeries including gastric lap band complicated by gastric perforation as well as cholecystectomy a year after her shunt was initially placed.

Patient Course

She was initially admitted with cellulitis and an abscess around an epigastric port site which crossed the VP shunt catheter. She underwent irrigation and drainage of the abscess as well as externalization of the VP shunt at her clavicle. Her hardware was removed completely, and she underwent an endoscopic third ventriculostomy. Abdominal wound catheter site culture grew Mycobacterium fortuitum. CSF remained negative on serial testing. She was treated with imipenem and doxycycline for five weeks, and then switched to oral azithromycin and doxycycline which was continued for three months. Doxycycline was changed to oral sulfamethoxazole-trimethoprim with azithromycin for another 1.5 months. She discontinued antibiotics at approximately six months due to nausea and intolerance. She continued to do well without any sign of infection.

## Discussion

NTM is a rare cause of infection in patients with neurosurgical hardware. We present two cases of serious hardware infection with NTM. These types of infections are rare, with only 19 cases reported (Table 1) [3-18]. Most cases involved infections with subgroups within the Mycobacterium fortuitum (48% of cases) and Mycobacterium abscessus (43% of cases). None of the patients were immunocompromised. The age range was 2 to 71 years, with a median age of 45 years. The majority of neurosurgical hardware involved was shunt catheters (70% of cases). Interestingly, 43% (9/21) of patients in the series and 64% (9/14) of patients with shunt hardware had symptoms of distal infection with peritonitis, pseudocyst or abscess formation. The majority of patients (86%; 18/21) had a positive CSF culture. The majority of device-related NTM infections are caused by Mycobacterium abscessus and Mycobacterium fortuitum, such as in our patients, whereas disseminated disease most commonly caused by Mycobacterium avium complex. Even with rapid hardware explantation and high-dose antibiotic therapy, mortality was 19% (4/21). 

**Table 1:** Overview of reported nontuberculous mycobacterial infections in patients with neurosurgical hardware VP, ventriculoperitoneal; P, lumbar-peritoneal; PEEK, polyetheretherketone; DBS, deep brain stimulation; EVD, external ventricular drain; IT, intrathecal; TMP/SMX,  trimethoprim-sulfamethoxazole; f/u, follow-up.

Author/year	Age/sex	Organism	Hardware	+ CSF	Treatment	Prognosis
Current study	25M	Mycobacterium abscessus	VP shunt, pseudocyst	Yes	Azithromycin, amikacin, cefoxitin, IT amikacin	Death, 1 month
	71F	Mycobacterium fortuitum	VP shunt, pseudocyst	No	Imipenem, doxycycline (5 weeks), azithromycin, bactrim	Stable, 8 months f/u
Zakrzewski et al., 2019 [3]	26F	Mycobacterium fortuitum	LP shunt	Yes	Amikacin, imipenem, moxifloxacin, TMP/SMX	Stable, 1 year f/u
Xess et al., 2019 [4]	14F	Mycobacterium fortuitum	VP shunt, pseudocyst	Yes	Linezolid, ofloxacin, clofazimine, clarithromycin	Stable, 3 months
Giovannenze et al., 2018 [5]	46M	Mycobacterium abscessus	PEEK implant	No	Amikacin, imipenem, linezolid (6 weeks)	Stable 12 months f/u
Moritz et al., 2017 [6]	62M	Mycobacterium goodii	DBS electrode	No	TMP/SMX, doxycycline (6 months)	Stable, 12 months f/u
Baidya et al., 2016 [7]	59M	Mycobacterium abscessus	VP shunt	Yes	Amikacin, clarithromycin, meropenem	Death, 1 month
Levy et al., 2016 [8]	67F	Mycobacterium abscessus	VP shunt, distal tubing in abdomen	Yes	Meropenem, amikacin, azithromycin	Death, 1 month
Montero et al., 2015 [9]	30M	Mycobacterium abscessus	VP shunt, signs of peritonitis	Yes	Amikacin, imipenem, azithromycin, IT amikacin	Stable, 4 years f/u
Cadena et al., 2014 [10]	14M	Mycobacterium fortuitum	VP shunt, abd pseudocyst	Yes	Meropenem, TMP-SMX, moxifloxacin (9 months)	Stable, 2 years f/u
Lee et al., 2012 [11]	49M	Mycobacterium abscessus	Ommaya	Yes	Amikacin, clarithromycin, imipenem, minocycline, minofloxacin	Stable, 1 year f/u
	19F	Mycobacterium abscessus	EVD	Yes	Amikacin, clarithromycin, imipenem, levofloxacin, moxifloxacin	Stable, 1 year f/u
	28F	Mycobacterium abscessus	Ommaya	Yes	Amikacin, cefoxitin, clarithromycin, linezolid, meropenem, moxifloxacin, tigecycline, TMP/SMX	Stable, 2 years f/u
Aliabadi et al., 2008 [12]	60M	Mycobacterium fortuitum	Baclofen pump	Yes	Amikacin, ciptro, clarithromycin, TMP/SMX (19 months)	
Uche et al., 2008 [13]	60F	Mycobacterium goodii	VP shunt	Yes	Imipenem, moxifloxacin	Stable, 3 months
Viswanathan et al., 2004 [14]	60F	Mycobacterium fortuitum	VP shunt, abdominal abscess	Yes	Kanamycin, ciprofloxacin	Stable, 6 months
Quinn et al., 2002 [15]	12M	Mycobacterium fortuitum	VP shunt, peritonitis	Yes	Amikacin, cefoxitin, clarithromycin, TMP/SMX	Stable, 14 months
Midani et al., 1999 [16]	13F	Mycobacterium fortuitum	VP shunt, retroperionteal abscess	Yes	Amikacin, TMP-SMX (6 months)	
Madaras-Kelly et al., 1999 [17]	45F	Mycobacterium fortuitum	IT pain pump	Yes	IV imipenem, ciprofloxacin, doxycycline, clarithromycin, 10 months of oral abx	Stable, 10 months
Flor et al,. 1996 [18]	2M	Mycobacterium gordonae	VP shunt, peritonitis	Yes	Isoniazid, ethambutol, streptomycin	Stable, unknown f/u
Chan et al., 1991 [19]	60F	Mycobacterium fortuitum	VA shunt	Yes	IT amikacin, amikacin, ofloxacin	Stable, 1 year f/u

The gold standard for diagnosis is AFB culture, which should be ordered based on clinical suspicion as it is often not routinely done. Our microbiology lab uses matrix-assisted laser desorption ionization time-of-flight (MALDI-TOF) technology to identify mycobacteria but the sensitivities take weeks to result at a reference lab.

Immediate surgical hardware removal must be performed. Antibiotic regimens follow American Thoracic Society guidelines on pulmonary NTM [5]. Treatments include parenteral therapy with amikacin and cefoxitin or imipenem with at least one oral agent for at least six months. If there is good clinical response, parenteral regimens are typically discontinued in four to six weeks [20]. Given its rarity, there is no single regimen established for the treatment of CNS infection but three to four drugs should be given until sensitivities become available. Mycobacterium abscessus complex comprises a group of multidrug-resistant NTM, and new treatment regimens are urgently needed [1].

The blood-brain barrier remains a unique problem. IT antibiotics with aminoglycosides may allow theoretically higher doses, and is a safe treatment option; however, there are no efficacy studies to date. Three cases from Table 1 utilized IT amikacin, of which CSF was effectively cleared in two.

## Conclusions

CNS hardware-related NTM infections are rare but are becoming more prevalent. Aggressive treatment should be pursued with immediate hardware removal, parenteral and oral antibiotics as well as IT antibiotics in severe cases. Clinicians should be aware of these organisms in patients with indolent and unusual presentations.
